# Emergency Department Visits for Heat-Related Emergency Conditions in the United States from 2008–2020

**DOI:** 10.3390/ijerph192214781

**Published:** 2022-11-10

**Authors:** Penelope Dring, Megan Armstrong, Robin Alexander, Henry Xiang

**Affiliations:** 1 College of Medicine, University of Cincinnati, Cincinnati, OH 45267, USA; 2 Center for Pediatric Trauma Research, Nationwide Children’s Hospital, Columbus, OH 43205, USA; 3 Center for Injury Research and Policy, Nationwide Children’s Hospital, Columbus, OH 43205, USA; 4 Center for Biostatistics, The Ohio State University, Columbus, OH 43210, USA; 5 College of Medicine, The Ohio State University, Columbus, OH 43210, USA; 6 College of Public Health, The Ohio State University, Columbus, OH 43210, USA

**Keywords:** emergency department, heat-related illness, climate change

## Abstract

Exposure to high temperatures is detrimental to human health. As climate change is expected to increase the frequency of extreme heat events, and raise ambient temperatures, an investigation into the trend of heat-related emergency department (ED) visits over the past decade is necessary to assess the human health impact of this growing public health crisis. ED visits were examined using the Nationwide Emergency Department Sample. Visits were included if the diagnostic field contained an ICD-9-CM or ICD-10-CM code specific to heat-related emergency conditions. Weighted counts were generated using the study design and weighting variables, to estimate the national burden of heat-related ED visits. A total of 1,078,432 weighted visits were included in this study. The annual incidence rate per 100,000 population increased by an average of 2.85% per year, ranging from 18.21 in 2009, to 32.34 in 2018. The total visit burden was greatest in the South (51.55%), with visits increasing to the greatest degree in the Midwest (8.52%). ED visit volume was greatest in July (29.79%), with visits increasing to the greatest degree in July (15.59%) and March (13.18%). An overall increase in heat-related ED visits for heat-related emergency conditions was found during the past decade across the United States, affecting patients in all regions and during all seasons.

## 1. Introduction

Scientists predict a global temperature increase exceeding 3 °C above pre-industrial averages by the end of the century [1]. A sharp increase in weather-related mortality is expected across the United States as a result of this climate change [2]. As temperatures rise, episodes of extreme heat are expected to become longer, more intense, and more frequent [3,4]. The negative impact of extreme heat events on human health, as it relates to both morbidity and mortality, has been well documented [5,6,7,8,9,10,11,12,13].

Emergency department (ED) patient volumes are usually seen to rise during episodes of extreme heat, which are typically defined as periods of temperature exceeding the 95th–99th percentile of region norms [5,6,14,15]. Unsurprisingly, visits for heat-related emergency conditions—a term used to encompass a spectrum of symptoms resulting from exposure to high temperatures [16]—are reported to increase by roughly 70% during these events [15,17]. Outside of extreme heat events, higher ambient temperatures are also harmful to human health [18]. Mortality risk is seen to rise by 8% for each degree (F) increase in maximum daily temperature from one day to the next [7]. Heat-related emergency conditions are also seen to increase on days with higher ambient temperature that do not meet the threshold of extreme heat, with one study citing a 393.3% excessive risk in heat-related ED visits for every 10° (F) increase in temperature [19].

Almost all investigations into heat-related ED visits have restricted the time course of analysis to extreme heat events during the warm season [15,19,20,21,22,23]. However, the effects of climate change have necessitated a new approach to researching heat-related emergency conditions. Ambient temperatures have risen to a greater degree during the winter months as compared to summer months, and seasonal shifts have seen warmer weather arrive earlier in the Spring and linger later into the Fall [24,25,26]. The concern for heat-related emergency conditions is therefore no longer just limited to the warm season, and should be evaluated throughout the year. Further, past investigations into heat-related ED visits have generally included narrow populations of interest, with studies focused on populations in California, Florida, and North Carolina [17,19,21,27]. Even the largest sample analyses, looking at all adult Medicare beneficiaries or the 14 CDC public health tracking states, captured only a quarter of the United States population.

At the time of our study, no article could be found describing the burden of heat-related emergency conditions in the United States on an annual and national scale over the past decade. This study aimed to characterize the national trends in ED visits for heat-related emergency conditions in United States from 2008–2020. Our hypothesis was that the incidence rate per 100,000 US population of heat-related ED visits increased significantly over the past decade.

## 2. Materials and Methods

ED records were obtained from the Nationwide Emergency Department Sample (NEDS), provided by the Agency for Healthcare Research and Quality as a part of the Healthcare Cost and Utilization Project [28]. The data provided by NEDS are de-identified and publicly available, so IRB approval was not required for this study. NEDS compiles information from both the State Inpatient Databases and State Emergency Department Databases, capturing patients that are seen in the ED and subsequently admitted to the hospital, transferred to another facility, or released following ED treatment. Data were available from NEDS for the years 2006–2020, but this analysis was restricted to 2008–2020 due to technical challenges in releasing the 2006–2007 NEDS dataset by the Agency for Healthcare Research and Quality. NEDS records were collected from 989 facilities that represent a 20-percent stratified sample of United States hospital-owned EDs [29].

ED visits were included in this study if the medical diagnosis, in any position, contained an International Classification of Diseases, Ninth Revision Clinical Modifications (ICD-9-CM) or Tenth Revision Clinical Modifications (ICD-10-CM) code for heat-related emergency conditions, as established by previous studies [30]. In the NEDS, ICD-9-CM codes were effective from the start of 2008 through quarter 3 of 2015, and included diagnostic codes 992.0–992.9, and external cause of injury codes E9000 and E9009. ICD-10-CM codes were effective from quarter 4 of 2015 through the end of 2020, and included codes T67.0–67.9, V932, X30, and X32. Diagnostic codes for accidents or exposure related to excessive man-made heat (E9001 and W92, respectively) were not included, as our analysis aimed to focus on natural heat related emergency conditions. See Appendix A for complete listing of ICD codes utilized in this study.

Within the NEDS dataset, there is a discharge weighting factor to use in generating national estimates. Weighted counts of heat-related ED visits were generated using the survey design stratum, cluster of hospitals, and weighing DISCWT method [28]. These weights, incorporating the survey design elements (strata and clustering), are applied to NEDS unweighted observations to generate national level frequency estimates. The Taylor series linearization method was used to estimate the standard error and confidence intervals of the weighted counts. We estimated the total counts for heat-related ED visits across the United States.

National temperature data were also collected for this study, to allow for a better understanding of the relationship between temperature and heat-related ED visits. Temperature data were obtained from the National Oceanic and Atmospheric Administration Climate at a Glance [31]. This data set compiles daily temperatures taken from each of the 344 climate divisions of the contiguous United States, and weighs divisional values by area to construct national averages [32]. For this analysis, data were gathered on an annual basis, as well as for the months of January, March, and July. January and July were chosen to evaluate the temperature trends in the coldest and hottest months of the year. March was subsequently included due to unanticipated results of large number of ED visits generated during data analysis for this month.

### 2.1. Study Variables

For heat-related ED visits included in this study, the date of the visit, hospital geographic location, patient sex, age, zip code income quartile, and payer status were analyzed. In this analysis, date of visit was recorded on a monthly time scale. Hospital geographic location was provided by NEDS as Northeast, South, Midwest, and West. As further geographic groupings were not possible due to the limited information provided by NEDS, these same stratifications were utilized during this analysis. Zip code income quartile was determined by NEDS using the median household income for the zip code attached to the patient visit. Payer status was listed by NEDS as public, private, self-pay, and other.

Patient age was provided in years. For the purposes of this study, age groups were generated using findings from previous works. Though inconsistent, studies have noted an increased risk among patients 0–4 years for heat-related ED visits [21], emergency medical services calls [33], and mortality [34]. Elderly patients have also been found to be more susceptible to heat-related emergency conditions [35]. An increase in heat-related ED visits has been observed in patients over 65 years old [21], and a stepwise increased risk of mortality has been seen in patients over 65, over 75, and over 85 [36]. Further, school aged children and younger adults are seen to face an increased risk for heat-related emergency conditions due to recreational and occupational exposures [22]. To best capture these findings, the groupings of patients as 0–4 years, 5–17 years, 18–49 years, 50–64 years, 65–79 years, and ≥80 years were used for subgroup analysis.

A small number of visits included in the study contained an ICD-9-CM or ICD-10-CM specific for heat-related emergency conditions, but did not have one or more of the analyzed variables. Hospital region, patient age, sex, or payer status was missing in less than 0.25% of weighted counts included in this study. Zip code income quartile was absent from 2.79% of included weighted counts.

### 2.2. Statistical Analysis

This study aimed to investigate the changes in the national incidence rate for heat-related emergency conditions from 2008–2020. Population data were utilized to generate crude incidence rates per 100,000 US population. Population data were from the Centers for Disease Control and Prevention WONDER online data set for Bridged-Race Population Estimates compiled by the National Center for Health Statistics [37]. This data set utilizes census information to estimate the total population of the United States on July 1st of each year, and was collected for the years 2008–2020 in this study. Percent change calculations were utilized to examine yearly shifts in incidence rates. Calculations were made on the general framework of [[(incidence rate 2020) − (incidence rate 2019)]/(incidence rate 2019)] × 100. Averages and 95% confidence intervals were generated for both the incidence rates and percent changes calculated across the study period.

This study also aimed to investigate the burden of heat-related ED visits, as stratified by the selected variables. Weighted counts of heat-related ED visits were used to compare the burden between subgroups. Percent change calculations were utilized to examine yearly shifts in weighted counts of heat-related ED visits, and were generated in the same method as described above. Averages and 95% confidence intervals were generated for both the weighted counts and percent changes calculated across the study period.

## 3. Results

Table 1 contains the national estimates for heat-related ED visits from 2008–2020, by hospital region, patient age, sex, zip code income quartile, and payer status. Over the study period, an estimated 1,078,432 ED visits for heat-related emergency medical conditions were recorded nationally.

The South (51.55%) accounted for the most heat-related ED visits over the study period, while the Northeast (10.17%) recorded the least. The Northeast (5.99%, 95% CI −21.14–33.12) and Midwest (8.52%, 95% CI −16.36–33.40) had the greatest average annual increase in heat-related ED visits from 2008–2020.

Patients aged 0–4 years (1.45%) and 5–17 years (10.84%) accounted for a lower percentage of heat-related ED visits than their population percentage (6.3% and 16.95%, respectively). Patients aged 18–49 years accounted for the most heat-related ED visits (53.54%), which represented a greater share than their population percentage (43.05%). Patients aged 50–64 years (19.08%), 65–79 years (10.53%), and ≥80 years (4.55%) all accounted for heat-related ED visit percentages similar to their population percentage (19.29%, 10.67%, and 3.74%, respectively).

Males (69.35%) accounted for the majority of heat-related ED visits during the study period.

Patients living in zip codes corresponding to the lowest income quartile had the greatest burden of heat-related ED visits (36.42%), while patients living in zip codes corresponding to the highest income quartile had the least (14.76%).

Patients utilizing public payer insurance accounted for the largest share of heat-related ED visits (36.30%).

**Figure 1:**
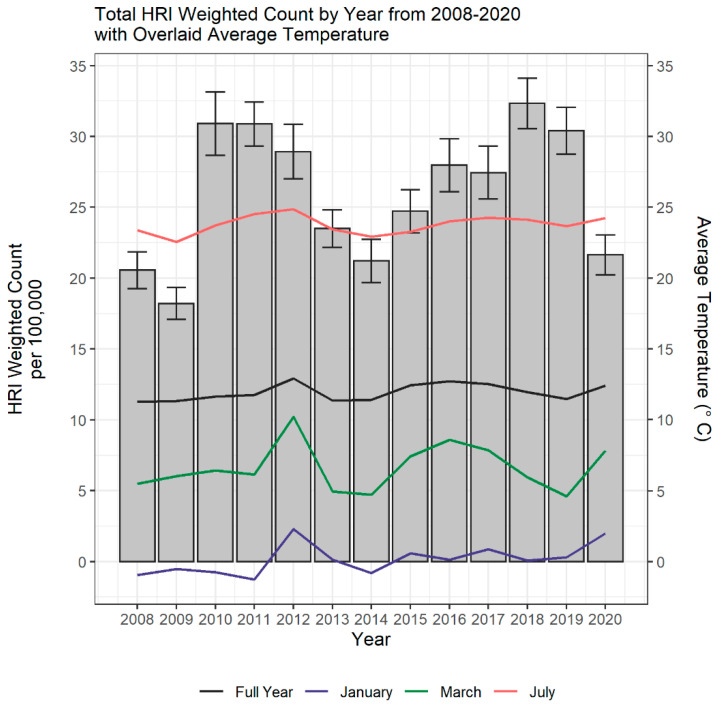
Heat-related ED visits per 100,000 population, in the United States from 2008–2020. Average national temperature (in Celsius) for the United States from 2008–2020.

Figure 1 displays the annual incidence rate of ED visits for heat-related emergency conditions per 100,000 US population from 2008–2020, with the corresponding average temperature across the same time period. The highest incidence rate was observed in 2018 (32.34 per 100,000), and the lowest incidence rate in 2009 (18.21 per 100,000). The average annual incidence rate for the study period was 26.06 (95% CI 23.41–28.70) per 100,000 and increased by an average of 2.85% (95% CI −11.43–17.13%) per year. The greatest increase in incidence rate (69.69%) was observed between 2009 (18.21 per 100,000) and 2010 (30.9 per 100,000). The largest decrease in incidence rate (−28.84%) was seen between 2019 (30.41 per 100,000) and 2020 (21.64 per 100,000).

The average annual temperature across the study period was 11.95 °C (95% CI 11.63–12.27), with the highest temperatures occurring in 2012 (12.93 °C), and the lowest in 2008 (11.27 °C). For the months of January, March, and July, the highest average temperatures were all observed in 2012 (2.34 °C, 10.23 °C, and 24.87 °C, respectively).

**Figure 2:**
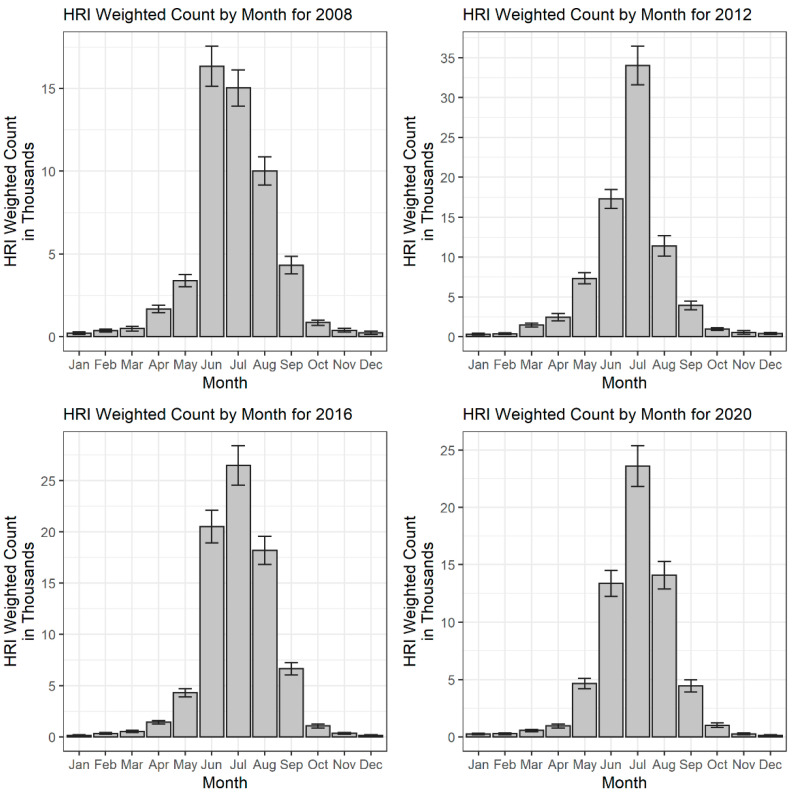
Heat-related ED visit weighted counts, stratified by month, in the United States for the years 2008, 2012, 2016, and 2020.

Figure 2 depicts the weighted counts for heat-related ED visits stratified by month of visit, for the years 2008, 2012, 2016, and 2020. These years were selected to observe changes in heat-related ED visits at even intervals across the study period. The warm season of May through September accounted for an average of 81.11% (95% CI 79.09–83.13%) of cases annually across the study period. July (29.79%) had the largest case volume over the study period, while December (0.31%) had the smallest. Across the study period, there was an average annual increase of 3.55% (95% CI −10.87–17.97%) per year in weighted counts of heat-related ED visits. July (15.59%, 95% CI −21.62–52.81%) and March (13.18%, 95% CI −17.84–44.20%) had the greatest average monthly increases.

## 4. Discussion

This study, based on weighted national medical records data, explored the trends in ED visits for heat-related emergency conditions across the United States from 2008 to 2020. An overall increase in the incidence rate for heat-related ED visits was observed during the study period, with this increase holding true both nationally and regionally. The burden of heat-related emergency conditions was seen to be greatest in the South. Most cases occurred during the month of July, with case numbers rising at the greatest rate in July and March.

While previous research on heat-related emergency conditions has largely been restricted to narrow populations of interest, the national approach of this study has allowed for a greater assessment of national trends and regional variations in heat-related ED visits across the United States. It is unsurprising that the South accounted for the greatest number of heat-related ED visits, knowing the regional temperature patterns of the United States. Looking at the demographic make-up of the South, this finding agrees with previous literature suggesting an increased risk of heat-related emergency medical conditions in more rural, non-White areas [23,38]. The observed increase in burden of heat-related ED visits is a particular cause for concern in the South, given the region’s workforce. In the coming years, Southern states are expected to face the greatest occupational risk to heat-related emergency conditions, and would most directly benefit from policies regulating workloads and shift times [39].

While the burden of heat-related ED visits was greatest in the South, the Northeast and Midwest had the greatest degree of increase in heat-related ED visits across the study period. This observed increase has several plausible explanations. On the basis of climate change, the Northeast has experienced greater warming and higher heat susceptibility as compared to the Southeast [40,41]. From a regional climate perspective, populations living in areas with significant seasonal weather variability are generally less able to adapt to higher temperatures. This is thought to be due to both physiology and the lesser availability of social adaptations, such as air conditioning [15,42]. Thus, focusing interventions on creating more social adaptations—particularly equipping public spaces with air conditioning and increasing tree cover—would be most effective in these regions [43].

When designing interventions for heat-related emergency conditions, it is important to note that the temperature threshold at which susceptibility increases is not the same across regions. Areas with lower average temperature have an increased risk for heat-related emergency conditions at lower temperature thresholds than areas of consistently warm climate [12]. While heat advisory alert systems have different temperature trigger points in different regions of the United States, past studies suggest the current models are not sufficient to both mitigate health risks and limit alarm fatigue [44]. Further research comparing heat advisory systems in different regions is warranted.

Results of this study also suggest that heat advisory trigger points should be evaluated during different months of the year. The burden pattern observed for the month of March suggest heat-related emergency medical risk increases with uncharacteristically high temperatures that are not exclusively limited to the warm season. A large peak of heat-related ED visits was observed in March 2012, which corresponds to the March 2012 heatwave [45]. Record breaking temperatures were recorded across the country, and the national monthly average temperature deviated nearly 5 °C above the twentieth century average [31]. Additional rises in heat-related ED visits were observed in March of 2015 and 2017, which similarly correspond to elevated temperatures in the western regions of the United States during these years [46,47]. As heat waves are predicted to begin earlier in the year [4], higher burdens of heat-related emergency conditions during March of subsequent years can be expected. This poses a particular concern, as extreme heat events in the early months of the year have been linked to greater health risks [48,49]. This is thought to be explained by the body’s lesser ability to adapt to higher temperatures following the winter months. Targeted interventions to limit heat-related emergency conditions enacted during the Spring could therefore be beneficial in the coming years.

### Limitations

Results of this study should be interpreted in the context of limitations. First, the methods used to identify heat-related ED visits did not allow for the distinguishment between primary, subsequent, or sequalae visits (within the ICD-9-CM codes). Thus, the counts reflected in this analysis may inaccurately overestimate the true count of unique patients experiencing heat-related emergency conditions within the population. However, given the course of illness and effectiveness of treatment for heat-related emergency conditions, it is reasonable to assume that the majority of included heat-related ED visits were primary visits caused by heat-related emergency conditions. Second, temperature data examined in this study was collected as a national average. This prevented the exploration into regional temperature patterns and gave rise to the potential for elevated temperatures in certain sectors of the United States to be obscured by depressed temperatures in other areas. Specific heat waves were also unable to be accounted for with these data. Finally, the nature of the ED data obtained from the Healthcare Cost and Utilization Project’s NEDS datasets prevented further patient information from being elicited, despite previous research suggesting an association between other demographic variables and heat-related ED visits. For example, it is therefore unknown if the experienced heat-related emergency medical condition was related to occupational or recreational activities. Examination into patient socioeconomic status was limited to the provided zip code median income and payer status. Race could not be evaluated in this study, as NEDS only included race for visits occurring in 2019 and 2020.

## 5. Conclusions

In this cross-sectional study, a significant increase in ED visits for heat-related emergency conditions was seen across the United States in the last decade, affecting populations of all ages, in all regions, and during all seasons. As the impact of climate change is predicted to worsen in the coming years, targeted interventions are necessary to mitigate the human health impact of this growing public health crisis.

## Figures and Tables

**Table 1:** Heat-related ED visit weighted counts, stratified by region, age, sex, income, and payer status in the United States from 2008–2020.

	2008	2009	2010	2011	2012	2013	2014	2015	2016	2017	2018	2019	2020
**Total**	62,519	55,868	95,580	96,215	90,789	74,276	67,542	79,273	90,364	89,216	105,660	99,832	71,298
**Hospital Region**													
Northeast	8010	5135	11,625	10,116	9969	11,846	5795	6879	8107	6850	10,700	8621	6057
Midwest	11,488	9758	21,560	28,646	27,704	16,122	11,213	14,054	17,838	15,452	21,049	17,634	14,126
South	30,059	28,405	50,697	44,491	38,902	31,288	36,149	44,470	50,889	46,118	56,728	59,604	38,151
West	12,961	12,571	11,698	12,961	14,214	15,021	14,384	13,870	13,531	20,796	17,183	13,974	12,964
**Patient Age**													
0–4	1161	1060	1390	1426	1615	1188	1487	1448	960	1067	1324	976	586
5–17	8432	7557	11,203	11,368	10,339	8589	7452	9244	9227	9512	10,721	9044	4242
18–49	33,515	29,284	52,416	52,571	48,905	39,973	36,299	44,063	49,370	45,759	54,015	52,609	38,657
50–64	10,585	9922	17,432	17,572	16,938	13,686	12,711	14,707	17,441	18,008	21,453	20,577	14,708
65–79	5648	5171	8730	8882	8992	7576	6853	6971	9411	10,280	12,964	12,393	9635
≥80	3178	2875	4403	4390	3995	3265	2735	2829	3955	4589	5184	4231	3466
**Patient Sex**													
Female	20,925	17,500	29,103	29,759	30,159	23,192	20,808	24,169	25,992	27,161	32,603	29,774	19,141
Male	41,573	38,288	66,444	66,413	60,627	51,078	46,724	55,104	64,360	62,056	73,041	70,047	52,147
**ZIP Code Income Quartile**													
1st (Lowest)	18,496	16,930	32,454	32,790	30,549	24,868	25,062	29,127	32,968	31,932	40,052	39,420	27,151
2nd	18,473	15,587	26,620	25,672	23,809	20,627	19,768	20,070	25,760	25,164	29,412	25,766	20,500
3rd	12,674	12,226	19,338	20,799	19,916	15,666	12,161	16,966	17,453	16,791	19,312	18,912	12,442
4th	10,603	9237	14,607	14,539	14,243	11,096	8523	11,141	11,985	12,671	13,993	13,174	8901
**Payer**													
Public	17,448	16,641	29,367	31,654	31,159	26,053	26,471	28,506	34,215	36,802	43,724	39,536	28,928
Private	24,787	21,392	34,472	33,654	30,949	24,123	21,248	27,067	29,206	27,818	32,600	30,248	20,644
Self-Pay	12,292	11,044	21,133	19,816	18,782	16,262	13,155	16,099	18,446	16,922	20,214	21,724	15,342
Other	7723	6674	10,281	10,623	9734	7714	6525	7405	8312	7348	8968	8221	6242

## Data Availability

Publicly available datasets were analyzed in this study. This data can be found here: https://www.hcup-us.ahrq.gov/nedsoverview.jsp (accessed on 1 November 2022).

## Appendix A

**Table A1:** ICD-9-CM and ICD-10-CM Codes to Identify Heat-Related ED Visits.

ICD-9-CM Code	Diagnosis
992.0	Heat stroke and sunstroke
992.1	Heat syncope
992.2	Heat cramps
992.3	Heat exhaustion, anhidrotic
992.4	Heat exhaustion, due to salt depletion
992.5	Heat exhaustion, unspecified
992.6	Heat fatigue, transient
992.7	Heat edema
992.8	Other specified heat effects
992.9	Unspecified effects of heat and light
E900.0	Accident caused by excessive heat due to weather conditions
E900.9	Accidents due to excessive heat of unspecified origin
**ICD-10-CM Code**	**Diagnosis**
T67.0	Heat stroke and sunstroke
T67.1	Heat syncope
T67.2	Heat cramps
T67.3	Heat exhaustion, anhidrotic
T67.4	Heat exhaustion, due to salt depletion
T67.5	Heat exhaustion, unspecified
T67.6	Heat fatigue, transient
T67.7	Heat edema
T67.8	Other specified heat effects
T67.9	Unspecified effects of heat and light
V93.2	Heat exposure on board watercraft
X30	Exposure to excessive natural heat
X32	Exposure to sunlight
